# Gitelman syndrome in a pediatric patient: a case report and literature review

**DOI:** 10.3389/fped.2025.1683867

**Published:** 2026-01-12

**Authors:** Tianhong Sun, Jing Yang, Jiajia Luo, Lina Ma

**Affiliations:** Pediatric Nephrology Department, Lanzhou University Second Hospital, Lanzhou, Gansu, China

**Keywords:** case report, Gitelman syndrome, hypokalemia, pediatrics, *SLC12A3* gene

## Abstract

**Objective:**

This article aimed to explore the clinical presentation, genetic underpinnings, and therapeutic approach to Gitelman syndrome (GS) in pediatric patients.

**Methods:**

This article presents a detailed case report of a child with persistent hypokalemia, incorporating clinical evaluations, laboratory testing, treatment strategy, and whole-exome sequencing. A literature review was conducted to contextualize the findings.

**Results:**

The patient was found to carry compound heterozygous mutations in the *SLC12A3* gene, with each inherited from a different parent. These mutations were identified as the primary cause of the child's refractory hypokalemia and impaired growth.

**Conclusion:**

Hypokalemia is a hallmark manifestation of pediatric GS. Genetic testing is instrumental for accurate diagnosis and differentiation from other hypokalemic conditions. The non-specific clinical phenotype of GS can lead to a missed or delayed diagnosis. In addition, co-occurrence of the p.T60M and p.T649M mutations is extremely rare in China. The presentation of this case underscores the need for heightened awareness of GS among pediatricians to enable early diagnosis and therapy, thereby optimizing the long-term quality of life of affected children.

## 1 Introduction

Gitelman syndrome (GS) is an uncommon autosomal-recessive disorder of renal tubular function, primarily resulting from mutations in the *SLC12A3* gene. This gene encodes the thiazide-sensitive sodium-chloride cotransporter (NCCT) located in the distal convoluted tubule (DCT) of the nephron (1, 2). Clinically, GS is characterized by chronic hypokalemia, hypomagnesemia, metabolic alkalosis, hypocalciuria, and compensatory hyperreninemic hyperaldosteronism (2). Due to its subtle and often non-specific symptoms, GS frequently eludes early detection, leading to underdiagnosis or misdiagnosis. Here we report a pediatric case of GS with chronic hypokalemia but normomagnesemia and without overt metabolic alkalosis. The patient presented to our department with growth retardation and QT-interval prolongation on electrocardiography (ECG). A genetic analysis revealed compound heterozygous mutations in *SLC12A3* that were inherited from both parents. The clinical symptoms improved after potassium supplementation. A comprehensive literature review is also provided to increase the rate of early recognition of GS in children and to reduce misdiagnoses.

## 2 Case presentation

### 2.1 Medical history and physical examination

#### 2.1.1 Presenting complaint

A 7-year-old Chinese boy was admitted to our hospital in October 2024 due to a 6-year history of documented hypokalemia, which had worsened over the previous month. At 1 year old, he had begun experiencing recurrent episodes of vomiting, and laboratory testing at the time revealed persistent hypokalemia (approximately 2.5 mmol/L). Despite multiple courses of potassium supplementation, his serum potassium level remained persistently low (2.5–2.9 mmol/L), but the condition was not medically followed up on. He developed recurrent nausea and vomiting 1 month prior to hospitalization, following an upper respiratory tract infection. At a local facility, his serum potassium level was 2.25 mmol/L. Despite receiving supportive therapy, including potassium supplementation and anti-infective treatment, his potassium level remained low (approximately 2.40 mmol/L), prompting referral for further evaluation. Throughout the course of his illness, he denied symptoms such as palpitations, fatigue, seizures, diarrhea, abdominal distension, or dysuria.

#### 2.1.2 Personal history

The patient is the third child (three pregnancies, three live births) and was delivered vaginally without complications or perinatal trauma.

As previous electrolyte panels had repeatedly shown the presence of hypokalemia, the family had routinely provided the patient with potassium-rich foods and fruits, such as potatoes, spinach, sweet potatoes, and bananas, in an attempt to correct the deficit through dietary measures.

#### 2.1.3 Family history

The patient's 12-year-old sister, 10-year-old brother, and both parents are reportedly healthy, with serum potassium and magnesium levels within normal ranges in all four family members. The parents are non-consanguineous.

#### 2.1.4 Physical examination

On admission, the patient's temperature was 36.2 °C, respiratory rate was 21 breaths/min, heart rate was 86 beats/min, and blood pressure was 96/70 mmHg. His height was 117.2 cm, and his weight was 18 kg, which were between the 3rd and 10th percentiles and below the 3rd percentile, respectively, based on standardized growth charts for Chinese males aged 0–18 years (3). A cardiovascular examination revealed normal heart sounds and rhythm. The patient’s abdomen was soft and non-tender, with normal bowel sounds (3/min). A neurological exam showed normal muscle strength and tone.

### 2.2 Supporting investigations

#### 2.2.1 Admission laboratory findings

On admission, the patient's serum potassium level was 2.10 mmol/L. Serum magnesium, blood pH, and urine pH levels were within normal limits, excluding metabolic alkalosis. Urinary potassium excretion over 24 h measured 82.27 mmol, which was near the upper threshold, suggesting renal potassium wasting. Plasma renin (PRA), angiotensin II (AII), and aldosterone (ALD) were significantly elevated in the supine position and rose further upon standing, consistent with activation of the renin-angiotensin-aldosterone system (RAAS). ECG revealed a prolonged QT interval, indicative of altered cardiac conduction due to chronic hypokalemia (details in Table 1).

**Table 1:** Laboratory and imaging findings on admission.

Test item	Item	Laboratory result	Reference range
Serum biochemistry	K^+^ (mmol/L)	2.10	3.7–5.2
	Mg^2+^ (mmol/L)	0.75	0.50–0.90
	Ca^2+^ (mmol/L)	2.42	2.1–2.8
	Na^+^ (mmol/L)	133.9	135–145
	Cl^−^ (mmol/L)	98.8	98–110
24-h urine electrolytes	K^+^ (mmol/24 h)	82.27	25–100
	Mg^2+^ (mmol/24 h)	3.01	3.0–5.0
	Ca^2+^ (mmol/24 h)	0.94	<1.8
	Na^+^ (mmol/24 h)	173.9	130–260
	Cl^−^ (mmol/24 h)	171.6	170–250
RAAS (supine position)	PRA (ng/mL/h)	9.86	0.15–2.33
	AII (pg/mL)	95.3	25.00–60.00
	ALD (pg/mL)	311.0	30.00–160.00
RAAS (upright position)	PRA (ng/mL/h)	16.61	0.10–6.56
	AII (pg/mL)	84.8	50.00–120.00
	ALD (pg/mL)	817.0	70.00–300.00
Fasting growth hormone levels	GH (ng/mL)	0.24	0.00–3.00
	IGF-1 (ng/mL)	51.50	40.00–255.00
	COR (µg/dL)	13.10	5.00–25.00
	ACTH (pg/mL)	8.03	5.00–46.00
	T3 (nmol/L)	1.93	1.33–3.08
	T4 (nmol/L)	165.7	76.81–137.91
	TSH (μIU/mL)	1.538	0.79–6.06
Urinalysis	Urine pH	7.0	4.5–8
Blood gas analysis	Blood pH	7.38	7.35–7.45
ECG	Prolonged QT interval §		
Renal ultrasound	Within normal limits §		

Note: PRA, Plasma renin activity; AII, Angiotensin II; ALD, Aldosterone; GH, Growth hormone; IGF-1, Insulin-like growth factor-1; COR, Cortisol; ACTH, Adrenocorticotropic hormone; T3, Triiodothyronine; T4, Tetraiodothyronine; TSH, Thyroid-stimulating hormone; RAAS, Renin-angiotensin-aldosterone system; ECG, Electrocardiogram.

### 2.3 Genetic testing results

**Figure 1:**
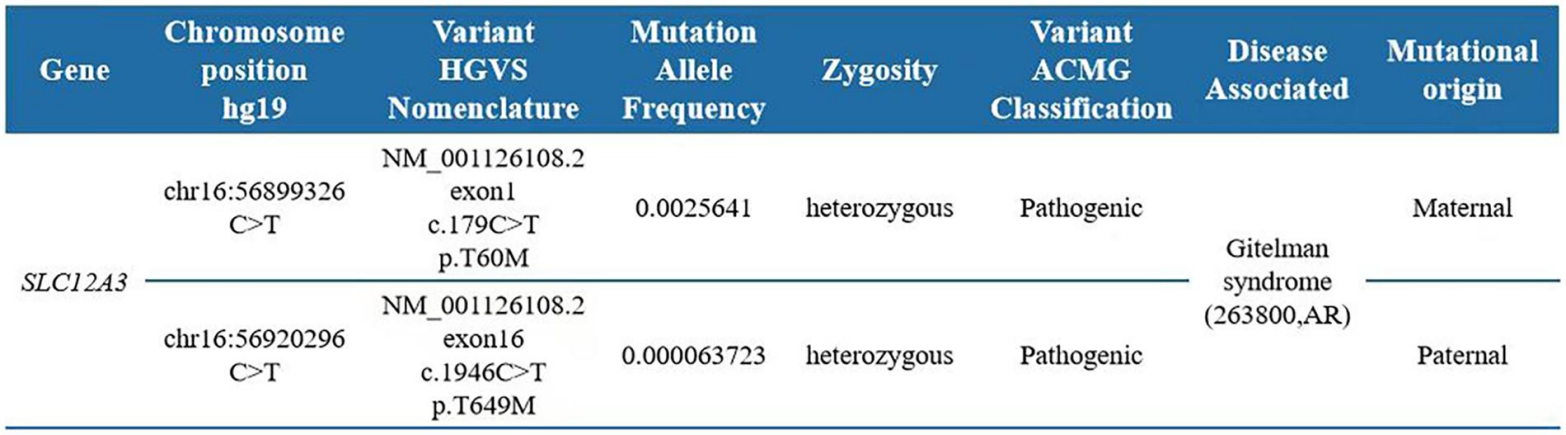
Pathogenic variants associated with the proband’s clinical information.

**Figure 2:**
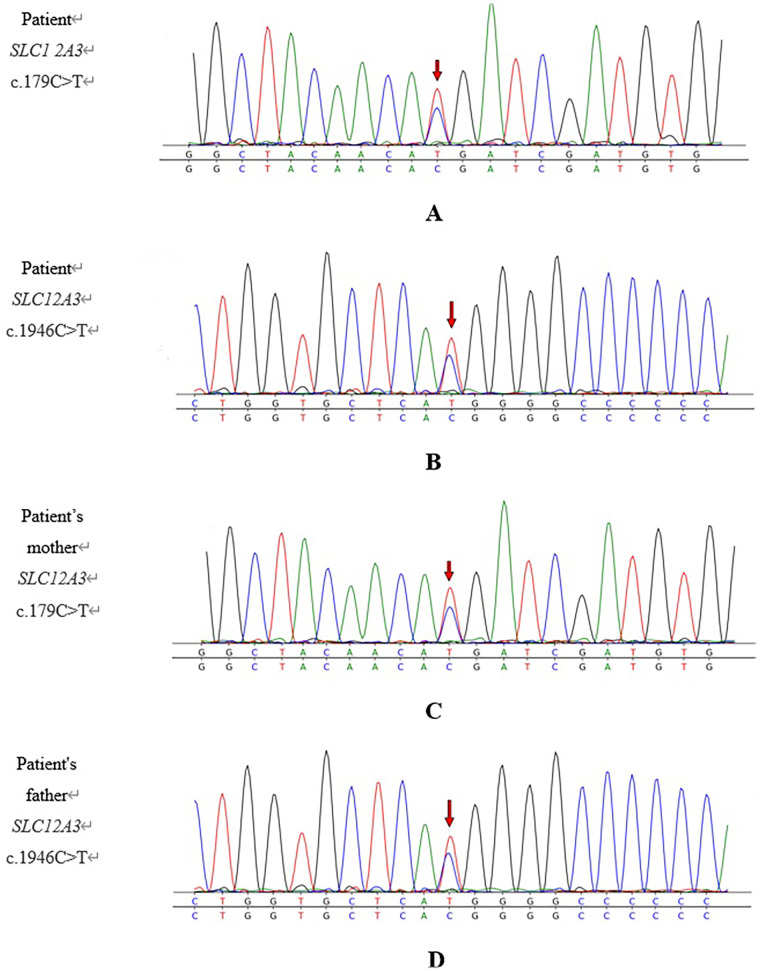
Sanger sequencing chromatograms for the *SLC12A3* loci present in the child and his parents. (**A**) Patient: *SLC12A3* c.179C>T; (**B**) patient: *SLC12A3* c.1946C>T; (**C**) patient's mother: *SLC12A3* c.179C>T; (**D**) patient's father: *SLC12A3* c.1946C>T. The variant site is marked with a red arrow. The child was found to harbor compound heterozygous variants in *SLC12A3*, namely, c.179C>T and c.1946C>T. The mother was found to be heterozygous for c.179C>T, and the father was heterozygous for c.1946C>T. The child had inherited one variant from each carrier parent.

After obtaining informed consent from the parents, whole-exome sequencing was performed. Whole-exome sequencing identified compound heterozygous variants in the *SLC12A3* gene, namely, c.179C > T (p.T60M) and c.1946C > T (p.T649M), based on the transcript NM_001126108.2. Interpretation using American College of Medical Genetics and Genomics (ACMG) guidelines classified both c.179C > T (PS3, PM2, PM3, and PP3 classifications) and c.1946C > T as pathogenic (PM2, PM3, and PP1 classifications). Both variants are recorded as disease-causing (DM) in the Human Gene Mutation Database (HGMD) and are inherited in an autosomal-recessive pattern. Parental testing confirmed that the c.179C > T mutation was maternally inherited, while the c.1946C > T mutation was paternally inherited. The combination of these mutations resulted in biallelic loss of gene function, consistent with the genetic basis of GS. Thus, the diagnosis was established based on the patient’s clinical presentation and the molecular findings (details in Figures 1, 2).

### 2.4 Treatment course

The treatment was administered in accordance with the 2017 Kidney Disease: Improving Global Outcomes (KDIGO) guidelines (4). After admission, intravenous potassium replacement was initiated with 40 mmol/day of potassium chloride, divided into two infusions. Within 48 h, the patient’s serum potassium levels rose from 2.1 mmol/L on admission to 2.88 mmol/L; the dose was therefore escalated to 100 mmol/day, given as four divided infusions. At discharge, the patient’s serum potassium level was 3.02 mmol/L and his nausea and vomiting had resolved. However, despite the improvement, his potassium level remained below the normal range, indicating the need for long-term potassium replacement on an outpatient basis. To avoid the disruption of daily life caused by frequent intravenous infusions, the therapy was converted to oral potassium chloride (granules plus sustained-release tablets). Considering the pharmacokinetics of enteral administration, the total daily dose was increased to 130 mmol, divided into four oral doses. No magnesium supplementation was instituted as serum magnesium levels had remained within the normal range. Over a 6-month follow-up period, the patient’s serum potassium level stabilized between 3.12 and 3.22 mmol/L, while his serum magnesium level fluctuated between 0.65 and 0.73 mmol/L and his acid–base balance remained within normal limits. His clinical symptoms, including nausea and vomiting, resolved completely. At discharge, repeat ECG showed a largely normal tracing. Six months later, the patient had gained 2 cm in height and 2 kg in weight (compared with only 1.8 cm and virtually no weight gain during the entire previous year), indicating an improved growth rate following potassium supplementation. Continued outpatient monitoring includes assessments of electrolyte balance, acid–base status, growth parameters, and surveillance for potential complications. Adjustments to treatment will be based on clinical course and laboratory markers. Table 2 shows the changes in serum potassium and magnesium levels and acid–base balance indices in the patient before treatment, after treatment, and over the 6-month follow-up period.

**Table 2:** Changes in serum potassium and magnesium levels and acid–base balance indices before treatment, after treatment, and during follow-up.

Date	Serum potassium level, mmol/L (reference range: 3.7–5.2 mmol/L)	Serum magnesium level, mmol/L (reference range: 0.5–0.9 mmol/L)	Blood pH (reference range: 7.35–7.45)	HCO_3_^−^, mmol/L (reference range: 21–28 mmol/L)	BE (reference range: −3.0 to 3.0)
5 October 2024 (admission)	2.10	0.75	7.42	25.6	2.8
7 October 2024	2.88	0.73	7.40	23.5	2.5
9 October 2024	2.96	0.74			
11 October 2024 (discharge)	3.02	0.72	7.37	21.9	2.3
20 November 2024	3.20	0.67			
15 December 2024	3.22	0.73	7.38	22.4	2.4
17 January 2025	3.20	0.69			
20 February 2025	3.17	0.65	7.39	23.1	2.6
16 March 2025	3.12	0.70			
12 April 2025	3.19	0.72	7.36	22.8	2.3

Serum biochemistry was analyzed using a Beckman AU5800 analyzer (Beckman Coulter Inc., Brea, CA, USA); serum potassium was determined using an ion-selective electrode and serum magnesium by colorimetry. The blood gas analysis was performed using a GEM Premier 5000 blood gas analyzer (Instrumentation Laboratory, Bedford, MA, USA).

Note: BE, Base excess.

Following treatment, the patient's serum potassium levels improved significantly, the nausea and vomiting resolved, and both height and weight increased markedly. These improvements substantially reduced the stress experienced by the patient and his family, who were greatly relieved that the symptoms had resolved and that the disease was now effectively controlled.

## 3 Discussion

GS is an uncommon autosomal-recessive renal tubular disorder that was first described by Gitelman, a member of the American Medical Association, in 1966 (5). Advances in molecular diagnostics, particularly genetic sequencing, have significantly expanded our understanding of this condition. Despite this progress, the onset of GS in children is often subtle, and many pediatric cases remain undiagnosed or are discovered incidentally during evaluation for unrelated health issues. Typical clinical manifestations in children are rarely reported, primarily because many cases are identified only after hypokalemia is noted during routine testing. In 1996, Simon et al. identified mutations in the *SLC12A3* gene, which encodes the renal NCCT involved in salt resorption that plays a key role in renal salt reabsorption within the DCT (6). Most patients exhibit compound heterozygous mutations, with each parent contributing a distinct mutation in the *SLC12A3* gene (7).

The *SLC12A3* gene, located on chromosome 16q13, encodes the thiazide-sensitive NCCT. Mutations in this gene disrupt the normal structure and/or function of the transporter, leading to impaired sodium and chloride reabsorption in the DCT (8, 9). As a result, more sodium reaches the collecting duct, increasing potassium excretion to maintain electroneutrality (10). This sodium loss also contributes to volume depletion, which in turn activates the RAAS. Aldosterone then enhances potassium secretion through the renal outer medullary potassium (ROMK) channels (11), exacerbating the hypokalemia. In addition, chronic potassium loss drives potassium ions into cells, which triggers a compensatory influx of hydrogen ions, leading to intracellular acidosis in renal tubular cells. This acidosis stimulates hydrogen ion secretion in both proximal and distal tubules. Elevated aldosterone levels from RAAS activation further potentiate this effect, resulting in metabolic alkalosis. In addition, hypokalemia stimulates H⁺–K⁺–ATPase activity in *α*-intercalated cells of the collecting duct, thereby promoting further hydrogen ion excretion and worsening the alkalosis (12, 13). Hypomagnesemia is another hallmark feature of GS. One proposed mechanism involves reduced activity or expression of TRPM6, a magnesium transport channel expressed on the apical membrane of DCT cells and in the duodenum. Studies in animal models suggest that structural atrophy of the distal tubule may also contribute to magnesium loss (14, 15). Persistent hypokalemia and hypomagnesemia can impair muscle and skeletal development in children, which may manifest as growth retardation. Furthermore, metabolic alkalosis may interfere with intracellular metabolic pathways, increasing the risk of skeletal abnormalities and osteoporosis. These electrolyte imbalances can also disrupt the growth hormone/insulin-like growth factor I (GH/IGF-I) axis, leading to short stature, delayed puberty, and impaired growth velocity in pediatric patients (13, 16). Prolonged QT intervals caused by hypokalemia further elevate the risk of potentially fatal arrhythmias (17).

Clinically, GS presents with highly variable and often non-specific symptoms, especially in pediatric patients. Many children do not display overt signs and are diagnosed only after incidental findings of hypokalemia. Commonly reported symptoms include a craving for salt, muscle weakness, dizziness, nocturia, polydipsia, fatigue, palpitations, and hypotension. In some cases, patients may experience syncope, polyuria, arthralgia, or febrile episodes. More severe or early-onset cases may present with chondrocalcinosis, QT-interval prolongation, ataxia, vertigo, carpopedal spasms, constipation, vomiting, and episodic paralysis. Less frequently observed symptoms include seizures, rhabdomyolysis, pseudotumor cerebri, blurred vision, and choroid calcification (4). Clinical studies indicate that early-onset GS (before 6 years old) is more likely to be associated with complications such as growth delay, chondrocalcinosis, spasms, rhabdomyolysis, and ventricular arrhythmias (4). In the presented case, while the onset of GS was early, diagnosis was markedly delayed due to its insidious onset. The child exhibited persistent vomiting and severe and treatment-resistant hypokalemia (serum potassium level consistently between 2.5 and 2.9 mmol/L despite supplementation). The child was initially seen by pediatricians who attributed the hypokalemia to vomiting, resulting in a significant delay in diagnosis. At admission to our hospital, the child was found to have not only severe hypokalemia (serum potassium level had declined to 2.1 mmol/L) but also growth retardation and QT-interval prolongation. These observations highlight the diagnostic challenges posed by the presentation of non-specific symptoms in young children. This case report aims to raise awareness among clinicians of this syndrome, thereby reducing missed or delayed diagnoses, ensuring the timely administration of standardized treatment, and minimizing complications.

Table 1 shows that although the patient’s fasting GH, IGF-1, cortisol (COR), and adrenocorticotropic hormone (ACTH) levels all fell within the reference ranges, the values were close to their lower limits, suggesting that the chronic hypokalemia may have negatively influenced the growth hormone axis. Likewise, while the indices reflecting acid–base balance were still within normal limits, they trended toward the upper end, especially on admission, implying that the long-standing hypokalemia had led to mild, persistent alkalosis. The patient had been hypokalemic for almost 6 years. The nausea and vomiting first appeared when his serum K⁺ level was 2.5 mmol/L, and subsequent measurements fluctuated between 2.5 and 2.9 mmol/L despite repeated supplementation; gastrointestinal symptoms were absent when the K⁺ level remained above 2.5 mmol/L. We therefore infer that a K⁺ level below 2.5 mmol/L represented an “alarm threshold” at which the child became symptomatic, prompting the family to seek medical care and the physician to prescribe additional potassium. The parents also increased dietary potassium during asymptomatic periods, which presumably maintained the K⁺ level above 2.5 mmol/L and prevented overt metabolic alkalosis. The patient experienced an upper-respiratory infection immediately before this admission, associated with a precipitous drop in serum potassium to 2.25 mmol/L, with recurrence of the nausea and vomiting. The slow rebound after supplemental potassium suggests that intermittent infections can markedly worsen and prolong hypokalemia in children with chronic GS. Admission ECG revealed QT-interval prolongation, indicating transient impairment of cardiac conduction by the hypokalemia. Restoration of normal tracing occurred after only a few days of intravenous potassium administration, supporting the view that the myocardium had not sustained permanent injury, most likely because intermittent supplementation and the consumption of potassium-rich food had provided partial protection throughout the chronic phase. This also explains why the patient’s skeletal muscle tone remained essentially normal; previous reports document myocyte damage when K⁺ is chronically <2.5 mmol/L (18). Finally, the patient’s serum magnesium level remained within the normal range, removing an additional factor that could have exacerbated hypokalemia, alkalosis, or muscular injury.

Differentiating GS from Bartter syndrome (BS) is essential, as both conditions involve inherited defects in renal salt handling and share overlapping clinical features such as hypokalemia, metabolic alkalosis, and secondary hyperaldosteronism (19). However, BS is genetically heterogeneous and comprises at least five subtypes, each associated with mutations affecting different transport proteins in either the DCT or the thick ascending limb of the loop of Henle (20). The application of genetic testing is therefore indispensable in accurately distinguishing between these syndromes and ensuring appropriate clinical management.

The therapeutic strategy for GS primarily involves correcting electrolyte disturbances, alleviating symptoms, and preventing long-term complications. Treatment typically involves lifelong potassium and/or magnesium supplementation tailored to the individual's needs (4). A target serum potassium level of ≥3.0 mmol/L and a magnesium level of ≥0.6 mmol/L are generally recommended. Potassium is commonly administered in the form of potassium chloride, while oral magnesium supplementation remains the first-line treatment for magnesium deficiency. Because magnesium deficiency can aggravate hypokalemia and hinder its correction, restoring magnesium levels is often a necessary first step, especially in cases of concurrent hypomagnesemia (4). Nutritional counseling should also encourage the consumption of foods rich in potassium and magnesium. Timely intervention is especially important in pediatric patients, as effective management can prevent electrolyte-related complications and support normal physical development.

Genetic testing in our patient identified the following two pathogenic missense mutations in *SLC12A3*: c.179C>T (p.T60M), which results in the substitution of threonine with methionine at amino acid position 60, and c.1946C>T (p.T649M), which causes the same amino acid change at position 649. Sanger sequencing confirmed that the patient inherited the c.179C>T mutation from his mother and the c.1946C>T mutation from his father, both of whom were clinically unaffected heterozygous carriers (Figure 2). The patient's compound heterozygous genotype aligns with the autosomal-recessive inheritance pattern characteristic of GS. While the p.T60M and p.D486N variants are most frequently encountered in Chinese populations (21), there are few reports of the p.T649M mutation identified in this case in China. A study by Zhang et al. (7), which reviewed 31 children with GS, observed only one child with both the p.T60M and p.T649M variants. The p.T60M mutation was paternally inherited, while the p.T649M mutation was inherited maternally along with an additional p.S748L mutation. The child was diagnosed at 11.9 years of age, presenting with fatigue, salt craving, and muscle weakness. The laboratory findings revealed hypokalemia, metabolic alkalosis, elevated plasma aldosterone concentration, and normal serum magnesium. Based on both this previous report and the present case, we infer that children with GS harboring concurrent p.T60M and p.T649M variants tend to have serum magnesium levels within the normal range and often exhibit atypical clinical features in the early stage. Loni et al. (22) reported a case of GS caused by a homozygous SLC12A3 variant (c.2309G > A, p.Gly770Asp). The patient became symptomatic at the age of 3 years, with severe manifestations, including refractory hypokalemia, hypomagnesemia, metabolic alkalosis, hypercalciuria, and muscle weakness. Electrocardiography revealed life-threatening wide QRS complexes and bradycardia. Growth retardation was also present. The child experienced multiple severe pulmonary infections. After aggressive antibiotic treatment, oxygen therapy, and correction of the hypokalemia and hypomagnesemia, the patient recovered. It can therefore be inferred that children harboring heterozygous GS mutations have milder clinical manifestations compared to those with homozygous mutations. However, the non-specific nature of these manifestations increases the difficulty of diagnosis. Moreover, young patients who become symptomatic at an early age are unable to articulate their discomfort, further raising the risk of a missed or delayed diagnosis.

In any case of persistent, unexplained hypokalemia, particularly in pediatric patients, GS should be considered in the differential diagnosis. Early genetic testing can facilitate a definitive diagnosis and timely intervention. Personalized treatment plans, continuous monitoring of electrolyte levels and renal function, and proactive management of complications are critical. Increasing awareness among healthcare providers is essential to improve early detection, initiate appropriate therapy, and enhance the long-term quality of life for pediatric patients with GS.

## Data Availability

The datasets presented in this study can be found in online repositories. The names of the repository/repositories and accession number(s) can be found in the article/Supplementary Material.
